# Structure-based design of antiviral drug candidates targeting the SARS-CoV-2 main protease

**DOI:** 10.1126/science.abb4489

**Published:** 2020-04-22

**Authors:** Wenhao Dai, Bing Zhang, Haixia Su, Jian Li, Yao Zhao, Xiong Xie, Zhenming Jin, Fengjiang Liu, Chunpu Li, You Li, Fang Bai, Haofeng Wang, Xi Cheng, Xiaobo Cen, Shulei Hu, Xiuna Yang, Jiang Wang, Xiang Liu, Gengfu Xiao, Hualiang Jiang, Zihe Rao, Lei-Ke Zhang, Yechun Xu, Haitao Yang, Hong Liu

**Affiliations:** 1 State Key Laboratory of Drug Research, CAS Key Laboratory of Receptor Research, Shanghai Institute of Materia Medica, Chinese Academy of Sciences, Shanghai 201203, China.; 2 School of Pharmacy, China Pharmaceutical University, Nanjing 210009, Jiangsu, China.; 3 Shanghai Institute for Advanced Immunochemical Studies and School of Life Science and Technology, ShanghaiTech University, Shanghai 201210, China.; 4 State Key Laboratory of Virology, Wuhan Institute of Virology, Center for Biosafety Mega-Science, Chinese Academy of Sciences, Wuhan 430071, China.; 5 College of Pharmacy, Nanjing University of Chinese Medicine, Qixia District, Nanjing 210023, China.; 6 National Chengdu Center for Safety Evaluation of Drugs, Westchina Hospital of Sichuan Unverisity, High-Tech Development Zone, Chengdu, Sichuan 610041, China.; 7 State Key Laboratory of Medicinal Chemical Biology, Frontiers Science Center for Cell Response, College of Life Sciences, College of Pharmacy, Nankai University, Tianjin 300353, China.

## Abstract

SARS-CoV-2 is the etiological agent responsible for the global COVID-19 outbreak. The main protease (M^pro^) of SARS-CoV-2 is a key enzyme that plays a pivotal role in mediating viral replication and transcription. We designed and synthesized two lead compounds (**11a** and **11b**) targeting M^pro^. Both exhibited excellent inhibitory activity and potent anti-SARS-CoV-2 infection activity. The X-ray crystal structures of SARS-CoV-2 M^pro^ in complex with **11a** or **11b**, both determined at 1.5 Å resolution, showed that the aldehyde groups of **11a** and **11b** are covalently bound to Cys145 of M^pro^. Both compounds showed good PK properties in vivo, and **11a** also exhibited low toxicity, suggesting that these compounds are promising drug candidates.

In late December 2019, a cluster of pneumonia cases caused by a novel coronavirus (CoV) was reported in Wuhan, China (*1*–*3*). Genomic sequencing showed that this pathogenic coronavirus is 96.2% identical to a bat coronavirus and shares 79.5% sequence identify to SARS-CoV (*4*–*6*). This novel coronavirus was named severe acute respiratory syndrome coronavirus 2 (SARS-CoV-2) by the International Committee on Taxonomy of Viruses, and the pneumonia was designated as COVID-19 by the World Health Organization (WHO) on February 11, 2020 (*7*). The epidemic spread rapidly to more than 212 countries and was announced as a global health emergency by WHO (*8*). No clinically effective vaccines or specific antiviral drugs are currently available for the prevention and treatment of COVID-19 infections. The combination of α-interferon and the anti-HIV drugs Lopinavir/Ritonavir (Kaletra®) has been used, but the curative effect remains very limited and there can be toxic side effects (*9*). Remdesivir, a broad-spectrum antiviral drug developed by Gilead Sciences, Inc., is also being explored for the treatment of COVID-19, but more data are needed to prove its efficacy (*10*–*12*). Specific anti-SARS-CoV-2 drugs with efficiency and safety are urgently needed.

A maximum likelihood tree based on the genomic sequence showed that the virus falls within the subgenus *Sarbecovirus* of the genus *Betacoronavirus (**6**)*. Coronaviruses are enveloped, positive-sense, single-stranded RNA viruses. The genomic RNA of CoVs is approximately 30 k nt in length with a 5′-cap structure and 3′-poly-A tail, and contains at least 6 open reading frames (ORFs) (*13*, *14*). The first ORF (ORF 1a/b), about two-third of genome length, directly translates two polyproteins: pp1a and pp1ab, because there is an a-1 frameshift between ORF1a and ORF1b. These polyproteins are processed by a main protease (M^pro^), also known as the 3C-like protease (3CL^pro^), and one or two papain-like proteases (PLPs), into 16 non-structural proteins (nsps). These nsps engage in the production of subgenomic RNAs that encode four main structural proteins (envelope (E), membrane (M), spike (S), and nucleocapsid (N) proteins) and other accessory proteins (*15*, *16*). Therefore, these proteases, especially M^pro^, play a vital role in the life cycle of coronavirus.

M^pro^ is a three-domain (domains I to III) cysteine protease involved in most maturation cleavage events within the precursor polyprotein (*17*–*19*). Active M^pro^ is a homodimer containing two protomers. The CoV M^pro^ features a non-canonical Cys-His dyad located in the cleft between domains I and II (*17*–*19*). M^pro^ is conserved among CoVs and several common features are shared among the substrates of M^pro^ in different CoVs. The amino acids in substrates from the N terminus to C terminus are numbered as fellows (-P4-P3-P2-P1↓P1′-P2′-P3′-), and the cleavage site is between the P1 and P1′. In particular, a Gln residue is almost always required in the P1 position of the substrates. There is no human homolog of M^pro^ which makes it an ideal antiviral target (*20*–*22*).

The active sites of M^pro^ are highly conserved among all CoV’s M^pro^s and are usually composed of four sites (S1′, S1, S2 and S4) (*22*). By analyzing the substrate-binding pocket of SARS-CoV M^pro^ (PDB ID: 2H2Z), novel inhibitors targeting the SARS-CoV-2 M^pro^ were designed and synthesized (Fig. 1). The thiol of a cysteine residue in the S1′ sites anchors inhibitors by a covalent linkage that is important for the inhibitors to maintain antiviral activity. In our design of new inhibitors, an aldehyde was selected as a new warhead in P1 in order to form a covalent bond with cysteine. The reported SARS-CoV M^pro^ inhibitors often have an (*S*)-γ-lactam ring that occupies the S1 site of M^pro^, and this ring was expected to be a good choice in P1 (*23*). Furthermore, the S2 site of coronavirus M^pro^ is usually large enough to accommodate the bigger P2 fragment. To test the importance of different ring systems, a cyclohexyl or 3-fluorophenyl were introduced in P2, with the fluorine expected to enhance activity. An indole group was introduced into P3 in order to form new hydrogen bonds with S4 and improve drug-like properties.

**Fig. 1:**
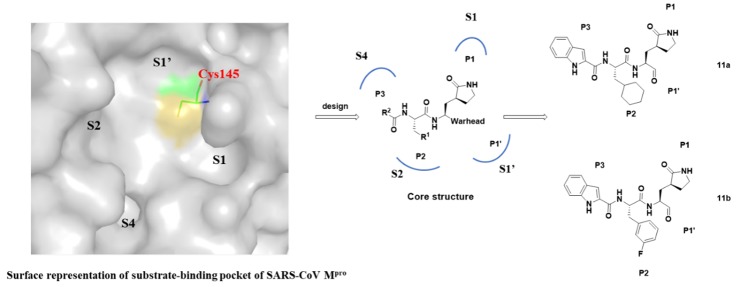
Design strategy of novel SARS-CoV-2 main protease inhibitors and the chemical structures of 11a and 11b.

The synthetic route and chemical structures of the compounds (**11a** and **11b**) are shown in scheme S1. The starting material (*N*-Boc-*L*-glutamic acid dimethyl ester **1**) was obtained from commercial suppliers and used without further purification to synthesize the key intermediate **3** according to the literature (*24*). The intermediates **6a** and **6b** were synthesized from **4** and acids **5a**, **5b**. Removal of the *t*-butoxycarbonyl group from **6a** and **6b** yielded **7a** and **7b**. Coupling **7a** and **7b** with the acid **8** yielded the esters **9a** and **9b**. The peptidomimetic aldehydes **11a** and **11b** were approached through a two-step route in which the ester derivatives **9** were first reduced with NaBH_4_ to generate the primary alcohols **10a** and **10b,** which were subsequently oxidized into aldehydes **11a** and **11b** with Dess-Martin Periodinane (DMP).

Recombinant SARS-CoV-2 M^pro^ was expressed and purified from *Escherichia coli* (*E. coli*) (*18*, *25*). A fluorescently labeled substrate, MCA-AVLQ↓SGFR-Lys (Dnp)-Lys-NH_2_, derived from the *N*-terminal auto-cleavage sequence from the viral protease was designed and synthesized for the enzymatic assay.

Both **11a** and **11b** exhibited high SARS-CoV-2 M^pro^ inhibition activity, which reached 100% for **11a** and 96% for **11b** at 1 μM, respectively. We used a fluorescence resonance energy transfer (FRET)-based cleavage assay to determine the IC_50_ values. The results revealed excellent inhibitory potency with IC_50_ values of 0.053 ± 0.005 μM and 0.040 ± 0.002 μM, for **11a** and **11b** respectively (Fig. 2).

**Fig. 2:**
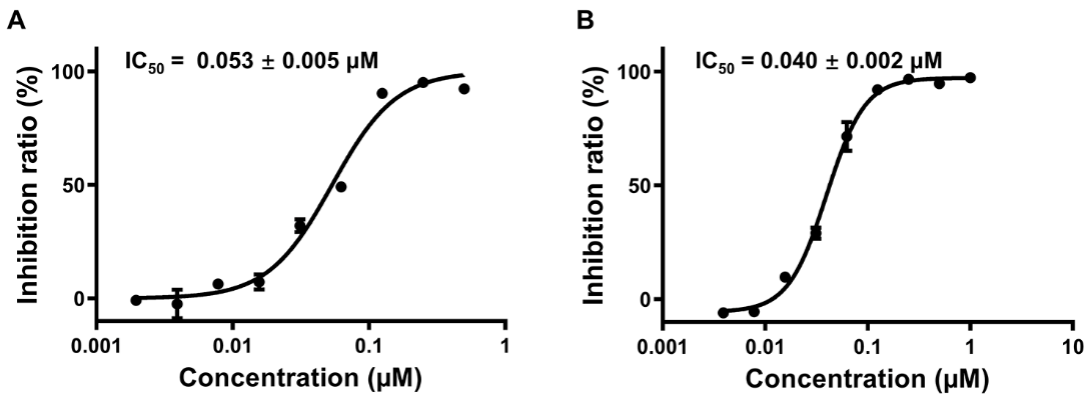
Inhibitory activity profiles of compounds 11a (A) and 11b (B) against SARS-CoV-2 M^pro^.

In order to elucidate the mechanism of inhibition of SARS-CoV-2 M^pro^ by **11a**, we determined the high-resolution crystal structure of this complex at 1.5-Å resolution (table S1). The crystal of M^pro^-**11a** belong to the space group *C2* and an asymmetric unit contains only one molecule (table S1). Two molecules (designated protomer A and protomer B) associate into a homodimer around a crystallographic 2-fold symmetry axis (fig. S2). The structure of each protomer contains three domains with the substrate-binding site located in the cleft between domain I and II. At the active site of SARS-CoV-2 M^pro^, Cys145 and His41 (Cys-His) form a catalytic dyad (fig. S2).

The electron density map clearly showed compound **11a** in the substrate binding pocket of SARS-CoV-2 M^pro^ in an extended conformation (Fig. 3A and fig. S3, A and B). Details of the interaction are shown in Fig. 3, B and C. The electron density shows that the C of the aldehyde group of **11a** and the catalytic site Cys145 of SARS-CoV-2 M^pro^ form a standard 1.8-Å C–S covalent bond. The oxygen atom of the aldehyde group also plays a crucial role in stabilizing the conformations of the inhibitor by forming a 2.9-Å hydrogen bond with the backbone of residues Cys145 in the S1′ site. The (*S*)-γ-lactam ring of **11a** at P1 fits well into the S1 site. The oxygen of the (*S*)-γ-lactam group forms a 2.7-Å hydrogen bond with the side chain of His163. The main chain of Phe140 and side chain of Glu166 also participate in stabilizing the (*S*)-γ-lactam ring by forming 3.2-Å and 3.0-Å hydrogen bonds with its NH group, respectively. In addition, the amide bonds on the chain of **11a** are hydrogen-bonded with the main chains of His164 (3.2 Å) and Glu166 (2.8 Å), respectively. The cyclohexyl moiety of **11a** at P2 deeply inserts into the S2 site, stacking with the imidazole ring of His41. The cyclohexyl group is also surrounded by the side chains of Met49, Tyr54, Met165, Asp187 and Arg188, producing extensive hydrophobic interactions. The indole group of **11a** at P3 is exposed to solvent (S4 site) and is stabilized by Glu166 through a 2.6-Å hydrogen bond. The side chains of residues Pro168 and Gln189 interact with the indole group of **11a** through hydrophobic interactions. Interestingly, multiple water molecules (named W1-W6) play an important role in binding **11a**. W1 interacts with the amide bonds of **11a** through a 2.9-Å hydrogen bond, whereas W2-6 form a number of hydrogen bonds with the aldehyde group of **11a** and the residues of Asn142, Gly143, Thr26, Thr25, His41 and Cys44, which contributes to stabilizing **11a** in the binding pocket.

**Fig. 3:**
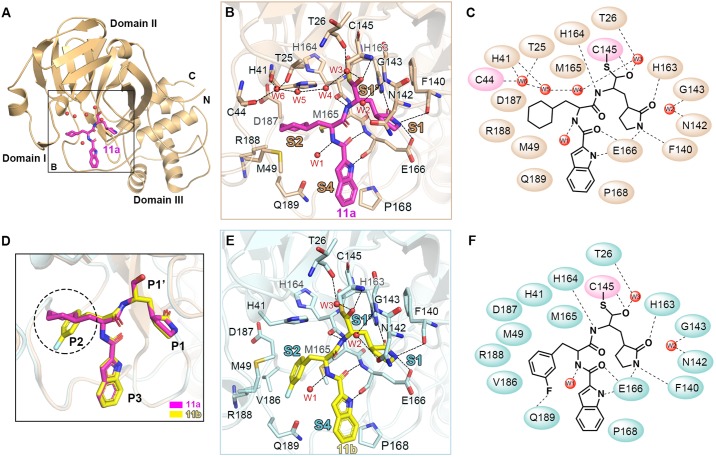
M^pro^-inhibitor binding modes for 11a and 11b. (**A**) Cartoon representation of the crystal structure of SARS-CoV-2 M^pro^ in complex with **11a**. The compound **11a** is shown as magenta sticks; water molecules shown as red spheres. (**B**) Close-up view of the **11a** binding pocket. Four subsites, S1′, S1, S2 and S4, are labeled. The residues involved in inhibitor binding are shown as wheat sticks. **11a** and water molecules are shown as magenta sticks and red spheres, respectively. Hydrogen bonds are indicated as dashed lines. (**C**) Schematic diagram of SARS-CoV-2 M^pro^-11a interactions shown in (B). (**D**) Comparison of the binding modes between **11a** and **11b** for SARS-CoV-2 M^pro^. The major differences between **11a** and **11b** are marked with dashed circles. The compounds of **11a** and **11b** are shown as magenta and yellow sticks, respectively. (**E**) Close-up view of the **11b** binding pocket. Hydrogen bonds are indicated as dashed lines. (**F**) Schematic diagram of SARS-CoV-2 M^pro^-11b interactions shown in (E).

The crystal structure of SARS-CoV-2 M^pro^ in complex with **11b** is very similar to that of the **11a** complex and shows a similar inhibitor binding mode (Fig. 3D and figs. S3, C and D, and S4A). The difference in binding mode is most probably due to the 3-fluorophenyl group of **11b** at P2. Compared with the cyclohexyl group in **11a**, the 3-fluorophenyl group undergoes a significant downward rotation (Fig. 3D). The side chains of residues His41, Met49, Met165, Val186, Asp187 and Arg188 interact with this aryl group through hydrophobic interactions and the side chain of Gln189 stabilizes the 3-fluorophenyl group with an additional 3.0-Å hydrogen bond (Fig. 3, E and F). In short, these two crystal structures reveal a similar inhibitory mechanism in which both compounds occupy the substrate-binding pocket and block the enzyme activity of SARS-CoV-2 M^pro^.

Compared with those of **N1**, **N3** and **N9** in SARS-CoV M^pro^ complex structures reported previously, the binding modes of **11a** and **11b** in SARS-CoV-2 M^pro^ complex structures are similar and the differences among these overall structures are small (Fig. 4 and fig. S4, B to F) (*22*). The differences mainly lie in the interactions at S1′, S2 and S4 subsites, possibly due to various sizes of functional groups at corresponding P1′, P2 and P4 sites in the inhibitors (Fig. 4, A and C).

**Fig. 4:**
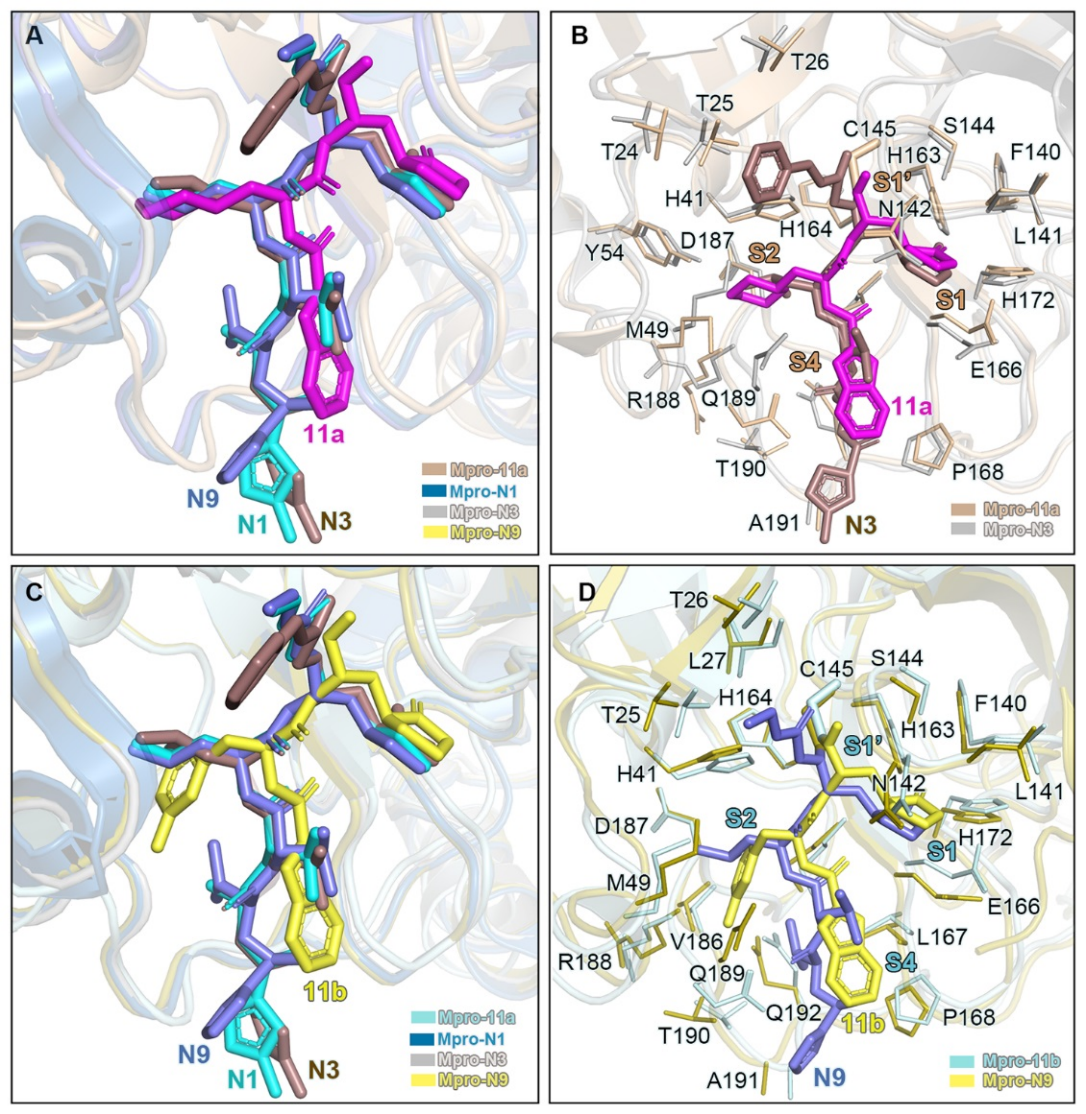
Comparison of the inhibitor binding modes in SARS-CoV and SARS-CoV-2 M^pro^s. (**A**) Comparison of binding modes of 11a in SARS-CoV-2 M^pro^ with those of N1, N3 and N9 in SARS-CoV M^pro^. SARS-CoV-2 M^pro^-11a (wheat, PDB code: 6LZE), SARS-CoV M^pro^-N1 (sky blue, PDB code:1WOF), SARS-CoV M^pro^-N3 (gray, PDB code: 2AMQ) and SARS-CoV M^pro^-N9 (olive, PDB code: 2AMD).11a, N1, N3 and N9 are shown in magenta, cyan, dirty violet and salt, respectively. (**B**) Comparison of the 11a and N3 binding pockets. Residues in M^pro^-11a structure and M^pro^-N3 structure are colored in wheat and gray, respectively. 11a and N3 are shown as sticks colored in magenta and dirty violet, respectively. (**C**) Comparison of binding modes of 11b in SARS-CoV-2 M^pro^ with those of N1, N3 and N9 in SARS-CoV M^pro^. SARS-CoV-2 M^pro^-11b (pale cyan, PDB code: 6M0K). 11b, N1, N3 and N9 are shown in yellow, cyan, dirty violet and salt, respectively. (**D**) Comparison of the 11b and N9 binding pockets. Residues in M^pro^-11b structure and M^pro^-N9 structure are colored in pale cyan and olive, respectively. 11b and N9 are shown as sticks colored in yellow and salt, respectively.

To further substantiate the enzyme inhibition results, we evaluated the ability of these compounds to inhibit SARS-CoV-2 in vitro (Fig. 5 and fig. S5). As shown in Fig. 5, compounds **11a** and **11b** exhibited good anti-SARS-CoV-2-infection activity in cell culture with EC_50_ values of 0.53 ± 0.01 μM and 0.72 ± 0.09 μM using plaque-reduction assay, respectively. Neither compound caused significant cytotoxicity, with half cytotoxic concentration (CC_50_) values of >100 μM, yielding selectivity indices (SI) for **11a** and **11b** of >189 and >139, respectively. Both immunofluorescence and quantitative real-time PCR were also employed to monitor the antiviral activity of **11a** and **11b**. The results show **11a** and **11b** exhibit a good antiviral effect on SARS-CoV-2 (Fig. 5 and fig. S5).

**Fig. 5:**
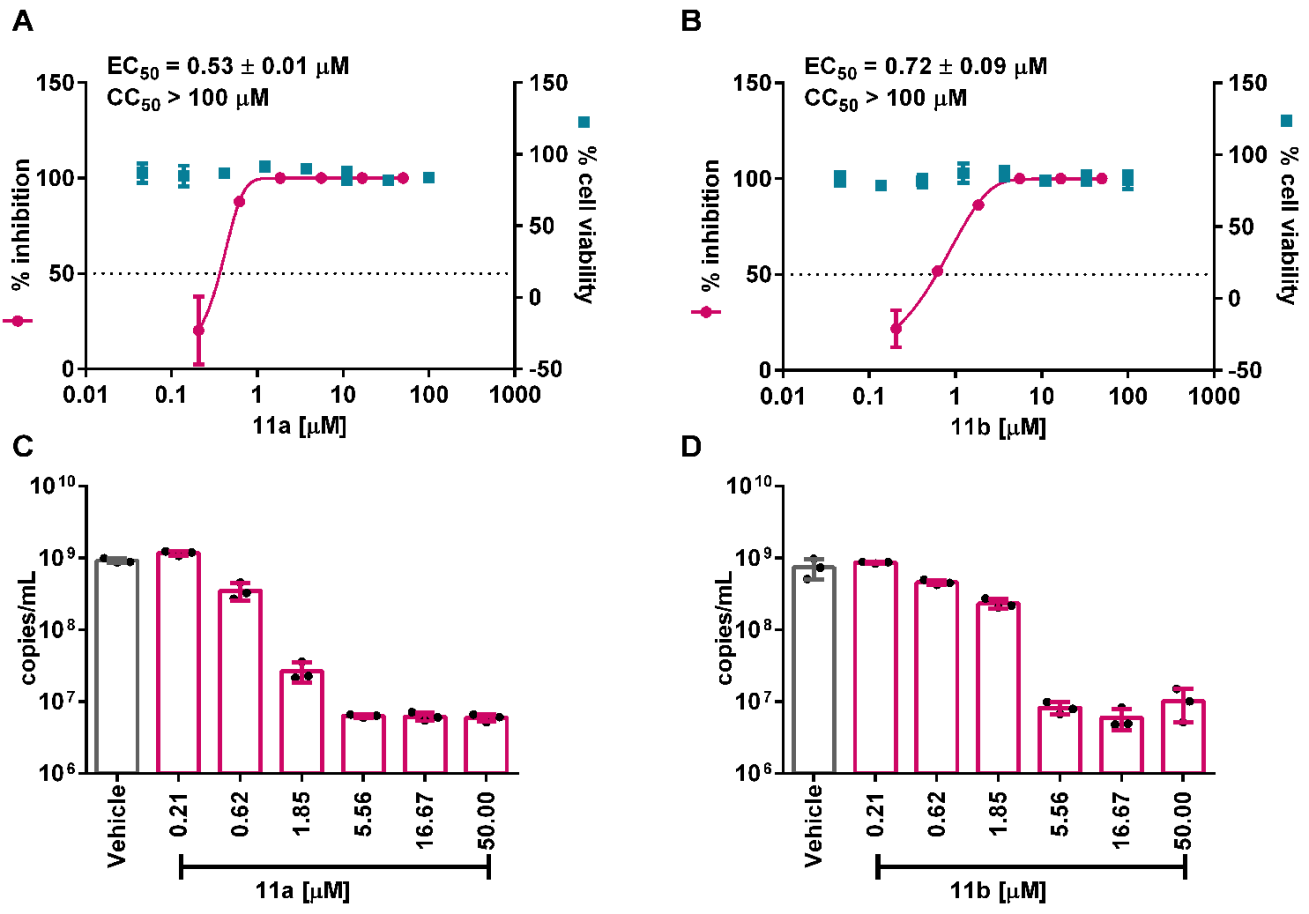
In vitro inhibition of viral main protease inhibitors against SARS-CoV-2. (**A** and **B**) Vero E6 cells were treated with a series concentration of indicated compounds **11a** and **11b** and infected with SARS-CoV-2 at an MOI of 0.05. At 24 hours post infection, viral yield in the cell supernatant was quantified by plaque assay. The cytotoxicity of these compounds in Vero E6 cells was also determined by using CCK8 assays. The left and right Y-axis of the graphs represent mean % inhibition of virus yield and mean % cytotoxicity of the drugs, respectively. **(C** and **D**) Viral RNA copy numbers in the cell supernatants were quantified by qRT-PCR. Data are mean ± SD, n = 3 biological replicates.

To explore the further druggability of the compounds **11a** and **11b**, both of the compounds were evaluated for their pharmacokinetic (PK) properties. As shown in table S2, compound **11a** given intraperitoneally (5 mg/kg) and intravenously (5 mg/kg) displayed a half-life (T_1/2_) of 4.27 hours and 4.41 hours, respectively, and a high maximal concentration (C_max_ = 2394 ng/mL) and a good bioavailability of 87.8% were observed when the compound **11a** was given intraperitoneally. Metabolic stability of **11a** in mice was also good (Clearance (CL) = 17.4 mL/min/mg). When administered intraperitoneally (20 mg/kg), subcutaneously (5 mg/kg) and intravenously (5 mg/kg), compound **11b** also showed good PK properties (the bioavailability of intraperitoneally and subcutaneously are more than 80%, and a longer T_1/2_ of 5.21 hours when **11b** was given intraperitoneally). Considering the danger of COVID-19, we selected the intravenous drip administration to further study for the reason that value of the area under the curve (AUC) is high and the effect is rapid. Compared with **11a** administrated intravenously, the T_1/2_ (1.65h) of **11b** is shorter and the clearance rate is faster (CL = 20.6 mL/min/mg). Compound **11a** was selected for further investigation with intravenous drip dosing in Sprague-Dawley (SD) rats and Beagle dogs. The results showed (table S3) that **11a** exhibited long T_1/2_ (SD rat, 7.6 hours and Beagle dog, 5.5h), low clearance rate (rat, 4.01 mL/min/kg and dog, 5.8 mL/min/kg) and high AUC value (rat, 41500 hours*ng/mL and dog, 14900 hours*ng/mL)). Those above PK results indicate that compound **11a** is worth to warrant further study.

An in vivo toxicity study (table S4) of **11a** has been carried out on SD rats and Beagle dogs. The acute toxicity of **11a** was measured on SD rats. No SD rats died after receiving 40 mg/kg by intravenous drip administration. When the dosage was raised to 60 mg/kg, one of four SD rats died. The dose range toxicity study of **11a** was conducted for seven days at dosing levels of 2, 6, and 18 mg/kg on SD rats and at 10-40 mg/kg on Beagle dogs. All animals received once daily dosing (QD), by intravenous drip, and all animals were clinically observed at least once a day. No obvious toxicity was observed in either group. These above data indicated that **11a** is good candidate for further clinical studies.

## Supplementary Materials

science.sciencemag.org/cgi/content/full/science.abb4489/DC1 Materials and Methods Scheme S1 Figs. S1 to S5 Tables S1 to S4 References (*26*–*29*)
